# Regulatory T lymphocytes and transforming growth factor beta in epithelial ovarian tumors-prognostic significance

**DOI:** 10.1186/s13048-015-0164-0

**Published:** 2015-06-17

**Authors:** Izabela Winkler, Barbara Wilczynska, Agnieszka Bojarska-Junak, Marek Gogacz, Aneta Adamiak, Krzysztof Postawski, Dorota Darmochwal-Kolarz, Tomasz Rechberger, Jacek Tabarkiewicz

**Affiliations:** II Department of Surgical Gynaecology, Medical University in Lublin, Jaczewski Street, 20-954 Lublin, Poland; Department of Clinical Immunology, Medical University in Lublin, Chodźki 4a Street, 20-093 Lublin, Poland; Department of Obstetrics and Perinatology, Medical University of Lublin, Jaczewski Street, 20-954 Lublin, Poland; Centre for Innovative Research in Medical and Natural Sciences, Medical Faculty of University of Rzeszów, Warzywna Street, 35-959 Rzeszów, Poland; Department of Paediatric Endocrinology and Diabetology with Endocrine-Metabolic Laboratory, Chodźki 2 Street, 20-093 Lublin, Poland

**Keywords:** Regulatory T lymphocyte, Transforming growth factor beta, Ovarian cancer, Benign tumor of ovary

## Abstract

**Background:**

Regulatory T lymphocytes (Treg) are characterized by the presence of CD4+ surface antigen. Today the transcription factor FOXP3 is considered to be the most specific marker of Treg cells. The aim of the study was to estimate the percentage of T_reg_ in peripheral blood and the tissue of the epithelial ovarian tumor and blood serum TGF-beta concentrations and relationships between them. Moreover, the aim of the study was to answer the question whether the percentage of Treg lymphocytes affects the time of survival in patients with ovarian cancer.

**Methods:**

The patients were divided into four groups, depending on the histopathological examination result: I – a group without any pathology within the ovaries (C; *n* = 20), II – a group with benign tumors (B; *n* = 25), III – with borderline tumors (BR; *n* = 11), IV – a group with cancer of the ovary (M; *n* = 24). The percentage of Treg lymphocytes in peripheral blood and the tissue was assessed using the flow cytometry method. TGF-beta cytokine concentration was estimated with the ELISA immunoenzymatic test. Statistical analysis of the results was conducted using the computer program Statistica 10.0PL (StatSoft, Inc).

**Results:**

No significant differences were found in percentages of Treg lymphocytes in peripheral blood between individual groups of patients (*p* = 0.11). However, we observed marked differences in the tissue of malignant and non-malignant tumors between individual groups of patients (*p* = 0.003). The analysis with the post hoc test revealed significantly higher TGF-beta concentration in the group of women with malignant tumors. Moreover, no relationship was found between TGF-beta concentration and the percentage of T_reg_ cells in peripheral blood and tumors of the ovary. No correlation was found between the percentage of Treg lymphocytes in peripheral blood (*p* = 0.4) and the tissue of ovarian tumors (*p* = 0.3) and the time of survival of patients with ovarian cancer.

**Conclusions:**

The recruitment of Treg lymphocytes toward the tumor is one of the mechanisms of escape of neoplasm from the response of the immune system. The percentage of Treg lymphocytes in peripheral blood and the neoplastic tissue does not influence the time of survival of patients with ovarian cancer.

## Background

The transforming growth factor-beta (TGF-beta), a pleiotropic cytokine, and regulatory T (T_reg_) cells play critical roles in suppressing the immune response. Treg cells were first described by Sakaguchi in 1995 [1]. They constitute 5-10 % of CD4+ T lymphocytes in peripheral blood [2, 3]. The phenotype of Treg cells is CD4 + CD25^high+^CTLA4 + GITR + FOXP3 + CD45RO + CD45RA-CD69-Ki-67 [4]. Treg cells are characterized by the presence of CD4+ surface antigen, typical o/f helper T lymphocytes. They also show expression of the α chain of the receptor for IL-2 (CD25^high+^). Today the transcription factor FOXP3 (*forkhead box P3*), which controls formation and differentiation of Treg cells, is considered to be the most specific marker of Treg cells. Mutations in the FOXP3 gene cause development of diseases based on autoaggression (e.g. type 1 diabetes), chronic inflammatory conditions and rejection of transplants [5].

Expression of the transcription factor FOXP3 is induced by TGF-beta in CD4 + CD25- lymphocytes, converting them into active regulatory T lymphocytes with the CD4 + CD25 + FOXP3+ phenotype [6]. It is also considered that T_reg_ lymphocytes thanks to their anchoring on the TGF-beta membrane induce expression of FOXP3 among the neighboring CD4 + CD25- cells as an effect of direct intercellular contacts [7, 8]. Andersson et al. claimed that inhibition of the T cell responds to conventional TGF-beta associated with the T_reg_ cell membrane and consequently it becomes the regulatory lymphocyte that inhibits further cell functions [9].

The elevation of T_reg_ in cancer suggests one mechanism by which tumors induce immunosuppression and this has been associated with a shorter overall survival. TGF-beta can increase as much as 4.5 times in patients with cancer compared to normal healthy patients and it could also be associated with a worse prognosis [10].

The study estimated the percentage of regulatory CD4 + CD25 + FOXP3+ T lymphocytes in peripheral blood and in healthy and neoplastic tissue in patients with benign, borderline and malignant epithelial tumors of the ovary. We also studied relationships between the percentage of regulatory CD4 + CD25 + FOXP3+ T lymphocytes and TGF-beta concentration in the microenvironment of benign and malignant ovarian tumors in comparison with the reference group. The question whether the percentage of Treg lymphocytes in malignant tumors correlates with the time of survival was also answered.

## Methods

### Studied material

The study comprised a group of 60 women hospitalized in II Clinic of Surgical Gynecology of the Medical University in Lublin. All the patients gave their written consent before participation in the study. The material for the study included peripheral blood, the tissue of a healthy ovary and tissues of malignant and non-malignant tumors of the ovary. The patients were divided into three groups: a group of 24 women with malignant epithelial tumors (*cystadenocarcinoma*) of the ovary (M), 25 women with benign tumors (*cystadenoma*) of the ovary (B), 11 women with borderline tumors (BR). All patients with ovarian cancer were in stage III or IV according to FIGO. The reference group included 20 women without any pathology within the ovaries (C) confirmed by histopathological examination.

### Isolation of mononuclear cells from peripheral blood

Mononuclear cells were isolated from peripheral blood immediately after the collection of samples from the cubital vein. The blood collected on EDTA was diluted (in 1:1 ratio) with 0.9 % buffered physiological saline solution (PBS). Mononuclear cells were isolated by centrifuging in density gradient with the use of Gradisol L preparation with specific weight of 1.077 g/ml (Aqua Medica, Łódź) for 20 min at a speed of 700 x g. The sediment containing peripheral blood mononuclear cells (PBMC) was rinsed twice in the PBS solution and the number and viability of cells were assessed (using the Neubauer chamber and the Trypan Blue Solution – 0.4 %, Sigma Aldrich, Germany, respectively). Viability below 95 % was the criterion disqualifying from conducting further study. The obtained mononuclear cells were suspended in RPMI 1640 culture medium with the addition of 20 % fetal calf serum (FCS) and 10 % dimethyl sulfoxide (DMSO, INC. Biomedicals, USA), and then frozen at −80 °C until the time of determination.

### Isolation of mononuclear cells infiltrating healthy and neoplastic tissue

Fragments of healthy or neoplastically changed tissue, sized at least 1 cm^3^, not containing necrotic areas, were collected during the surgical procedure. They were subsequently cut into small pieces using a scalpel. The minced tissues were suspended in 30 ml of RPMI 1640 medium (cat. no. F1215, Biochrom, USA) and digested in the mixture containing: 1 mg/ml of type IA collagenase (cat. no. C9891, Sigma-Aldrich, USA), 1 mg/ml type I DNA-ase (cat. no. DN25-1G, Sigma-Aldrich, USA), 0.1 mg/ml hyaluronidase (cat. no. H3506-100MG, Sigma-Aldrich, USA) at the temperature 37 °C for 60 min, being continuously stirred. After digestion the obtained suspension was filtered through a sieve (70 μm, BD Biosciences, USA) and centrifuged for 5 min at a speed of 700 x g. The obtained cells were rinsed twice in RPMI 1640.

### Estimation of the percentage of regulatory T lymphocytes

Estimation of the percentage of regulatory T lymphocytes among peripheral blood mononuclear cells and in healthy and neoplastic tissue was made with the flow cytometry method using the Human Treg Flow™ Kit (FOXP3 Alexa Fluor® 488/CD4 PE-Cy5/CD25 PE, BioLegend®, USA) with the use of FACSCanto apparatus (BD Biosciences, USA).

### Assessment of the TGF-beta cytokine concentration in blood serum

TGF-beta concentration in blood serum was determined using the ELISA method. The Quantikine® Human TGF-beta Immunoassay (R&D System, USA) with the fidelity <4.6 pg/ml was used for determination. The examination procedure was performed according to the manufacturer’s recommendations, and the automatic reader VICTOR3 (Perkin Elmer, USA) was used to interpret the results. The computer program WorkOut2 2.0 cooperating with the reader and basing on the known standards concentrations produced logarithm or half-logarithm linear curves on the basis of which it calculated concentration of the cytokine in the examined samples.

### Statistical analysis

Statistical analysis of the results included calculation of the values: mean (M), median (Med), minimum (min), maximum (max), standard deviation (SD) and 95 % confidence interval (c). Most of the examined features did not have normal arrangement, therefore non-parametric tests were used in order to verify statistical hypotheses:

- Kruskal-Wallis test – to verify differences between more than two groups
- post hoc (Dunn’s) test – to assess internal differences between two groups
- Wilcoxon test- to assess differences between the percentage of T_reg_ in peripheral blood and tissue
- Spearman’s rank correlation coefficient and its significance test – to assess relationships between two parameters.

Kaplan-Meier analysis which enabled us to draw survival curves depending on the interval of the Treg percentage values was also performed. The results whose significance level was not greater than 0.05 were accepted as statistically significant. Statistical analysis of the results was conducted using the computer program Statistica 10.0PL (StatSoft, Inc).

## Results

### Characteristics of the studied groups

The studied groups did not differ with reference to age, BMI, the number of deliveries and concentration of leukocytes in the blood (Table 1). However, significant differences were found in the time interval between the first and the last menstruation (*p* = 0.02). Significant differences occurred between the groups of women with malignant tumors and borderline tumors (M vs. BR; 5.2 mg/ml vs. 1.3 mg/ml; *p* = 0.03).

**Table 1:** Demographic and clinical characteristics of the studied groups

Group of patients	Without pathology	Benign tumor	Borderline tumor	Malignant tumor	p (Test Kruskal-Wallis)
	(C)	(B)	(BR)	(M)	
	N = 20	N = 25	N = 11	N = 24	
Parameters	Median	Median	Median	Median	
	(Min-Max)	(Min-Max)	(Min-Max)	(Min-Max)	
Age (years)	50 (37–78)	55 (32–85)	47 (35–77)	55 (44–80)	0.4
BMI (kg/m^2^)	28 (21–36.7)	26 (11.9-46.7)	31.6 (17.9-45.7)	25.6 (25–35.2)	0.053
Parity (n)	2 (0–4)	2 (0–7)	2 (0–5)	2 (0–6)	0.46
Time from menarche to menopause (years)	33.5 (25–42)	34 (18–45)	32 (13–38)	36 (28–42)	0.02
Leukocytes 10^3^cells/μl	7.2 (4.17-11.8)	5.8 (3–21)	7.2 (5–10.8)	7.3 (3.58-13.5)	0.29

### Estimation of the percentage of regulatory T lymphocytes in peripheral blood

**Fig. 1:**
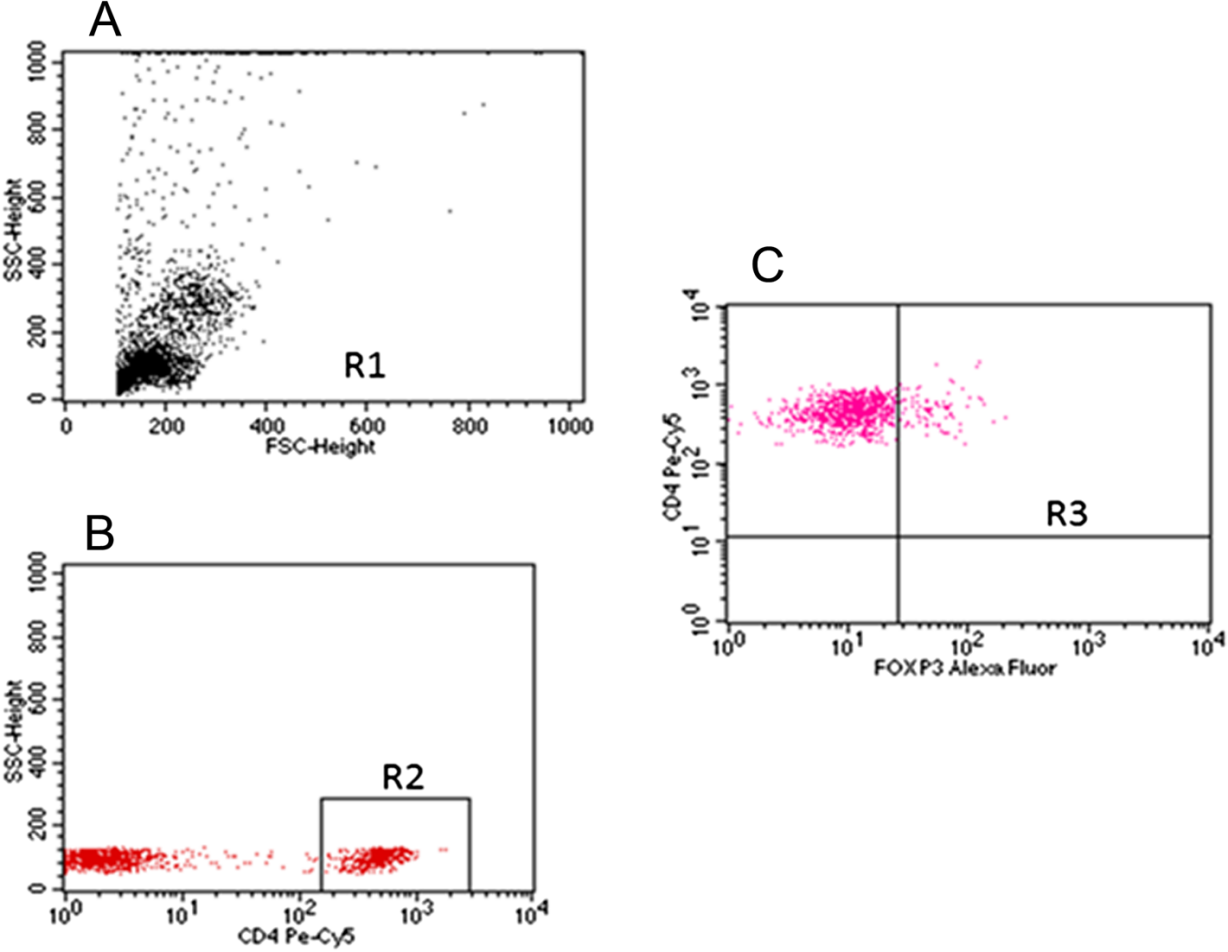
Sample cytometric analysis of T_reg_ lymphocytes in peripheral blood. **a**- Assessment of percentage of mononuclear cells (R1) based on FSC and SSC. **b**- Assessment of percentage of CD4+ cells using anti-CD4 Pe-Cy5 antibody (R2). **c**- Assessment of percentage of CD25 + FOXP3+ lymphocytes (R3) among CD4+ cells

**Fig. 2:**
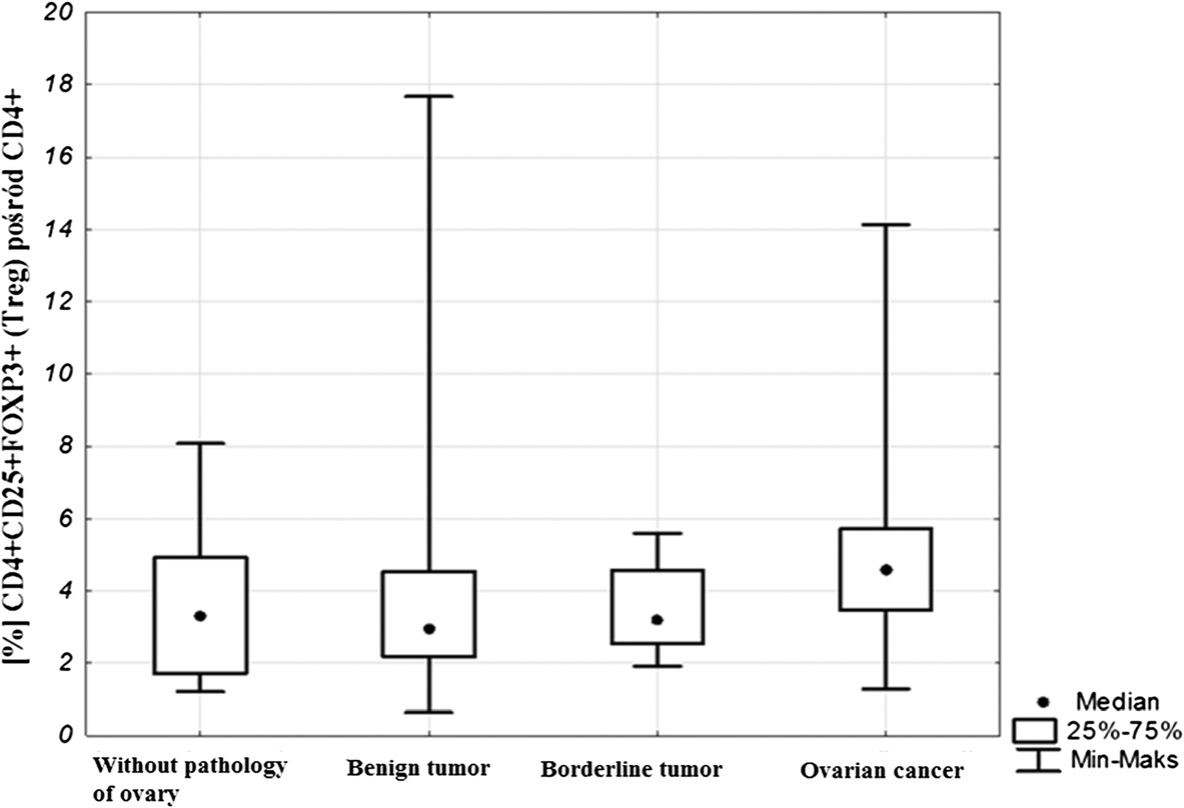
Percentage of lymphocytes CD4 + CD25 + FOXP3+ in peripheral blood of benign and malignant tumors of the ovary

No significant difference was found with reference to the percentage of CD4 + CD25 + FOXP3 + (T_reg_) lymphocytes in peripheral blood between the studied groups of women (Kruskal-Wallis test; *p* = 0.11). The post hoc test analysis did not reveal differences between the individual groups either. The percentage median of Treg lymphocytes in the studied groups was as follows: in the group of women without ovarian pathology it was 3.31 %, in the group with non-malignant tumors 2.94 %, with borderline tumors 3.19 % and in the group of women with malignant tumors 4.57 % (Figs. 1 and 2).

### Percentage of regulatory T lymphocytes (CD4 + CD25 + FOXP3+) in the tissue of (malignant and non-malignant tumors of) the ovary

**Fig. 3:**
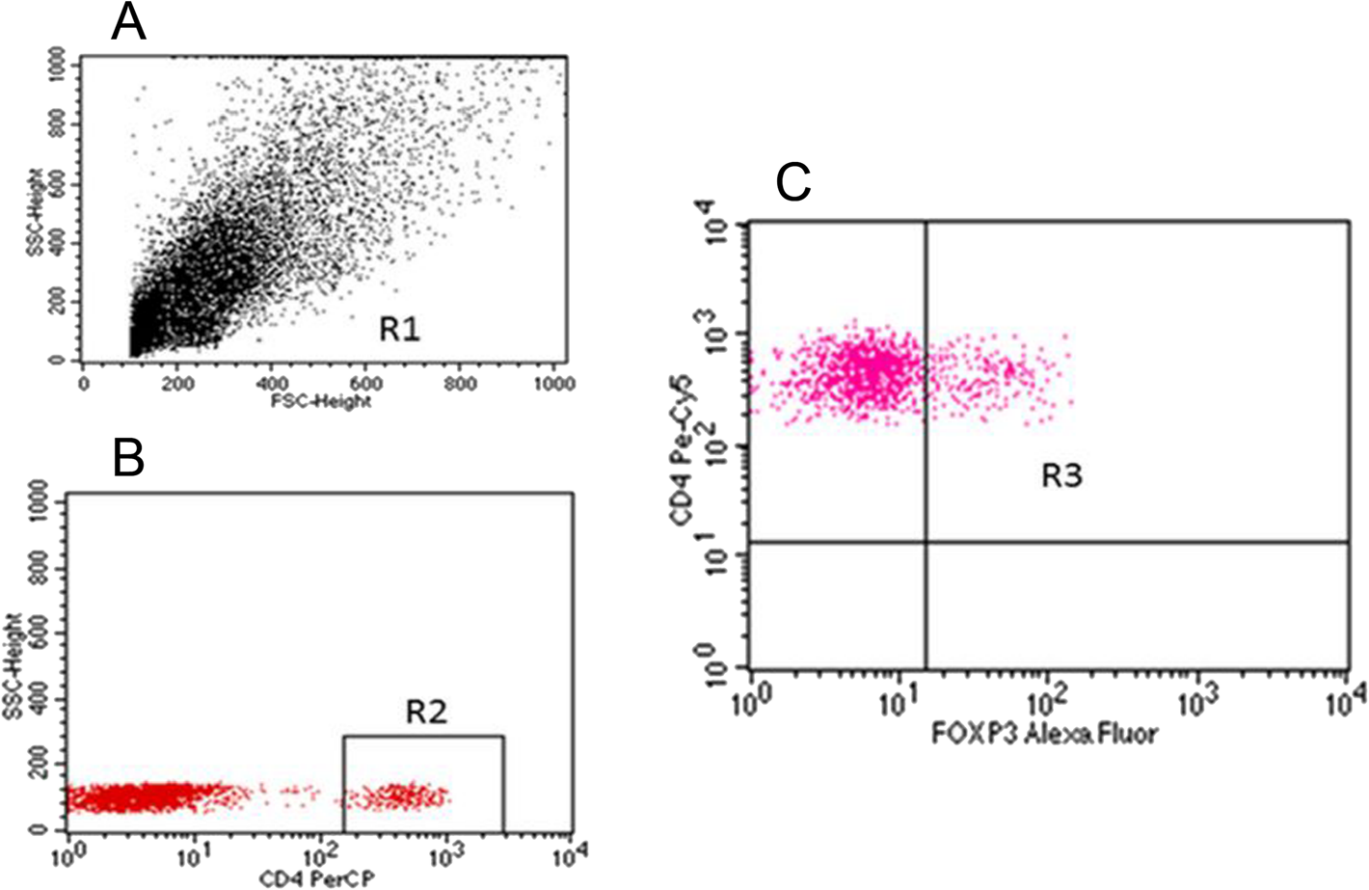
Sample analysis of the percentage of T_reg_ cells in the tissue of tumor. **a**- Assessment of mononuclear cells (R1) based on FSC and SSC. **b**- Assessment of percentage of CD4+ cells (R2). **c**- Assessment of percentage of CD25 + FOXP3+ lymphocytes (R3) among CD4+ cells

**Fig. 4:**
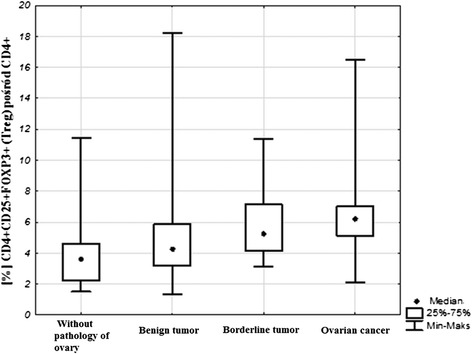
Percentage of lymphocytes CD4 + CD25 + FOXP3+ in tissue of benign and malignant tumors of the ovary

The percentage of Treg cells in the tissue without ovarian pathology was 3.59 %, in the group of women with non-malignant tumors it was 4.25 %, among the patients with borderline tumors 5.26 % and in the group of women with malignant tumors 6.21 % (Figs. 3 and 4). Significant differences were found with reference to the percentage of CD4 + CD25 + FOXP3+ Treg lymphocytes in malignant and non-malignant tumors between individual groups of patients (Kruskal-Wallis test; *p* = 0.003). Significantly higher values of Treg lymphocytes were observed in the group of women with malignancies in comparison to the group without ovarian pathology: (M) 6.21 vs. (C) 3.59; post hoc test; *p* = 0.002). No significant difference was found in the percentage of CD4 + CD25 + FOXP3+ Treg lymphocytes between the remaining groups of patients: control and non-malignant tumors (C) 3.59 vs. (BR) 5.26; post hoc test; *p* = 0.16; (C) 3.59 vs. (B) 4.25; post hoc test; *p* = 0.85) and between the groups with non-malignant tumors (BR) 5.26 vs. (B) 4.25; post hoc test; *p* = 1.0). There were no significant differences either observed between the groups with malignant and non-malignant tumors (M) 6.21 vs. (B) 4.25; post hoc test; *p* = 0.15; (M) 6.21 vs. (BR) 5.26; post hoc test; *p* = 1.0).

### Relationships between the percentage of Treg lymphocytes in peripheral blood and in the ovarian tissue of malignant and non-malignant tumors

Statistical analysis using Wilcoxon pair test showed significant relationships between the percentage of Treg lymphocytes in peripheral blood and the percentage of Treg cells in benign (*p* = 0.00001), borderline (*p* = 0.003) and malignant (*p* = 0.0007) tissue of tumors.

### Prognostic assessment of the percentage of T_reg_ lymphocytes in peripheral blood in malignant tumors

**Fig. 5:**
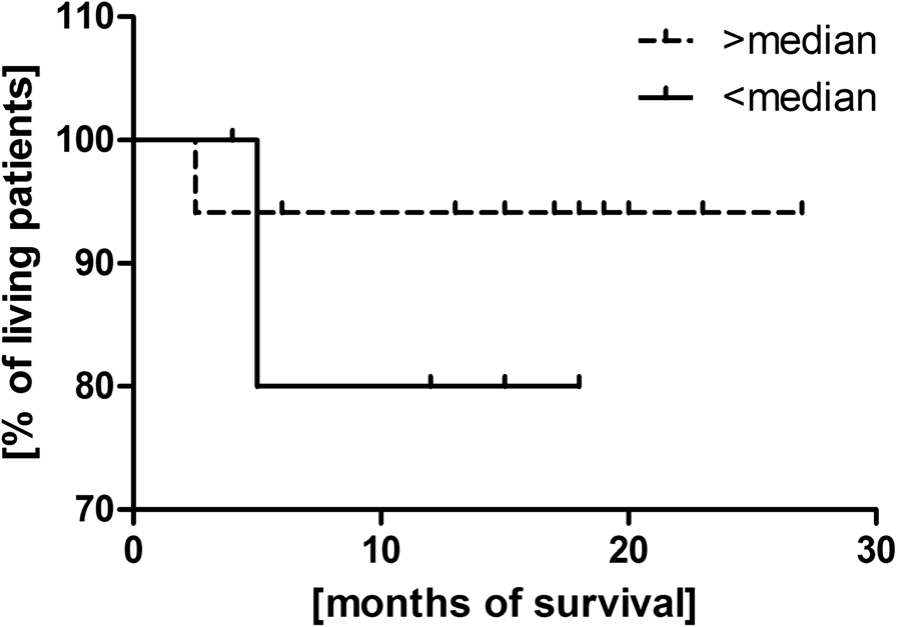
Kaplan-Meier survival curve in relation to the percentage of T_reg_ in peripheral blood in ovarian cancer

Kaplan-Meier survival analysis was performed in the group of patients with ovarian cancer in relation to the value of the T_reg_ percentage in peripheral blood. The patients were divided into two groups depending on the median value (>3.7 % or <3.7 %) obtained from the patients results in all the groups. A survival probability curve was obtained in the two groups formed in relation to the median value of the T_reg_ percentage. Mantel-Cox test produced the insignificant result *p* = 0.4 (Fig. 5).

### Prognostic assessment of T_reg_ lymphocytes in the tissue of patients with ovarian cancer

**Fig. 6:**
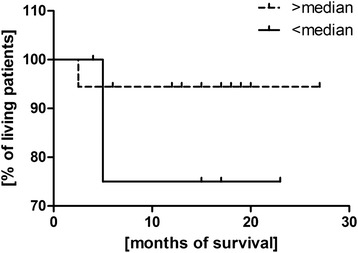
Kaplan-Meier survival curve in relation to the percentage of T_reg_ in the tissue of ovarian cancer

Kaplan-Meier survival analysis was performed in the group of patients with ovarian cancer. The patients were divided into two groups depending on the median value (>4.6 % or <4.6 %) obtained from the patients’ results in all the groups. A survival probability curve was obtained in the two groups formed in relation to the median value of the T_reg_ percentage (Fig. 6). Mantel-Cox test produced the insignificant result *p* = 0.3.

### Blood serum TGF-beta concentration in women with malignant and non-malignant tumors

**Fig. 7:**
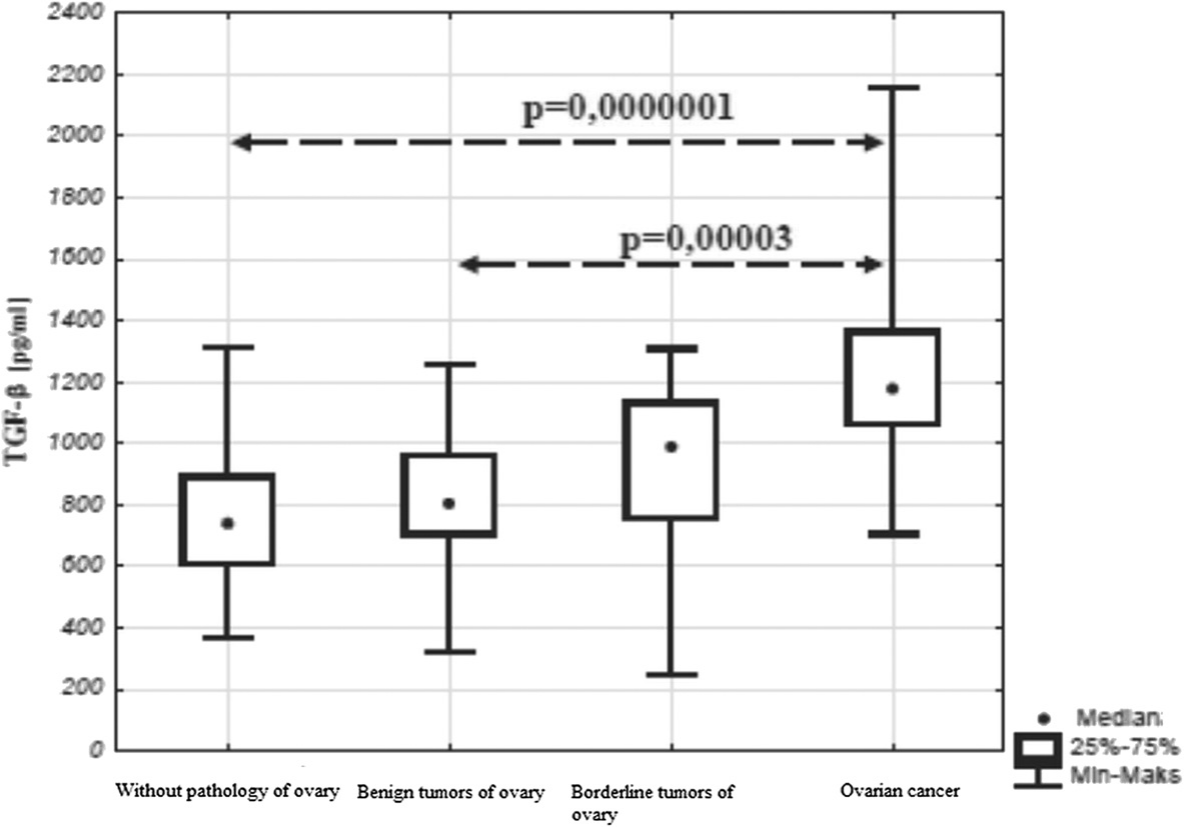
Concentration of TGF- beta in serum in benign and malignant tumors of the ovary

The mean concentration of TGF-beta in ovarian cancer was 1243.3 pg/ml, in the group with benign tumors it was 835.1 pg/ml, in borderline tumors - 908.3 pg/ml (Fig. 7). Statistical analysis using Kruskal-Wallis test revealed statistically significant differences between the analyzed groups (*p* = 0.00001). Further analysis with the post hoc test showed significantly higher TGF-beta concentration in the group of women with ovarian cancer in comparison to the control group (M vs. C; *p* = 0.0000001) and to the group with benign tumors (M vs. B; *p* = 0.00003). No significant differences were found between the control group and the group with non-malignant tumors (C vs. B; *p* = 1.0; C vs. BR; *p* = 0.32) and between the groups with borderline tumors and malignant tumors (BR vs. M; *p* = 0.06). Moreover, there were no significant differences observed between the groups with benign and borderline tumors (B vs. BR; *p* = 1.0).

### Relationships between TGF-beta concentration in blood serum and the percentage of T_reg_ in peripheral blood and the tissue of tumors

The values of the Spearman’s rank correlation coefficient revealed no significant relationships between the T_reg_ percentage in ovarian tumors and TGF-beta concentration (Table 2) in peripheral blood and the tissue of ovarian tumors.

**Table 2:** Relationships between TGF-β concentration in blood serum and the T_reg_ percentage in peripheral blood and the tissue of ovarian tumors

Parameters	Without pathology of ovary	Benign tumor	Borderline tumor	Ovarian cancer
Relationships between TGF-beta and T_reg_ percentage in peripheral blood	r_s_ = 0.03	r_s_ = −0.18	r_s_ = −0.29	r_s_ = 0.32
	*p* = 0.87	*p* = 0.37	*p* = 0.4	*p* = 0.13
Relationships between TGF-beta and T_reg_ percentage in the tissue of ovarian tumors	r_s_ = 0.09	r_s_ = −0.09	r_s_ = −0.07	r_s_ = 0.05
	*p* = 0.69	*p* = 0.64	*p* = 0.83	*p* = 0.8

## Discussion

Cancer of the ovary is number one cause of death among neoplasms of the reproductive organs. Despite a breakthrough view of histogenesis of ovarian cancer suggested by Dubeau, formation of a new classification of ovarian cancer by Kurman, great involvement and work of many researchers, a significant breakthrough in anticancer therapy has not been achieved. This neoplasm is most frequently diagnosed at the advanced stage of the disease, when the chances of survival decrease from 93 % at stage I to approximately 10 % at stage IV. It is estimated that the mean percentage of 5-year survival in ovarian cancer is 30 % [11–15]. The basic management strategy towards patients with ovarian cancer is still surgery complemented with chemotherapy. A lack of effective methods used in screening examinations, late diagnosis and increasing resistance to chemotherapeutic treatment cause us to seek new directions in prevention, detection and treatment of ovarian cancer. In order to decrease mortality researchers try to introduce new methods of target therapy based on immunotherapy.

The texture of a neoplasm creates unique conditions for its development. The neoplastic microenvironment induces abnormal interactions between the cells of the tumor and the host. Due to its role in invasion of the tumor and formation of metastases this environment is the subject of intense research. Apart from cancer cells it contains competent immune cells, such as macrophages and lymphocytes. Among the subpopulation of lymphocytes the basic elements of the neoplastic compartment are regulatory T cells (Treg) and these are potential tools for immunotherapy.

In our studies the percentage of Treg lymphocytes among CD4+ T lymphocytes was assessed in two compartments: in peripheral blood and in the microenvironment of the neoplastic tissue of non-malignant and malignant ovarian tumors. They were identified on the basis of the presence of CD4 + CD25+ surface markers and the intracellular factor FOXP3 which plays a decisive role in the process of maturation and regulation of the Treg cells function. The subject of the research seems interesting as, since 1995 when Sakaguchi et al. [1] discovered these lymphocytes, their increased numbers have been found in the course of neoplastic diseases. Woo et al. [16] observed an increased percentage of CD4 + CD25+ cells in the population of lymphocytes infiltrating the tumor in the cases of non-small cell lung carcinoma and ovarian cancer. Fialova et al. [17] also noticed that the number of Treg cells in the ovarian cancer tissue was greater than in peripheral blood. This is confirmed by our studies as the percentage of Treg lymphocytes was the highest in the group with ovarian cancer and differed significantly from other groups with non-malignant tumors and without ovarian pathology. Woo et al. [16] also found that Treg lymphocytes release TGF-beta cytokine, which confirmed their suppressor function in the course of neoplastic diseases. Further study conducted by Liyanage et al. [18] showed an increased number of CD4 + CD25+ cells not only within the microenvironment of the tumor but also in peripheral blood of patients with breast and pancreatic cancer. Moreover, researchers found that an increased level of Treg in cancer patients has a systemic character. Our studies did not confirm this observation. The examined groups did not differ in the percentage of Treg in peripheral blood, however, their percentage value was the highest in the group of patients with ovarian cancer. On the other hand, our research found significant relationships between the Treg percentage in peripheral blood and in the tissue of non-malignant and malignant ovarian tumors, which may be related to migration of Treg toward the tumor. This preferential movement toward and concentration in the tissue of an ovarian tumor were also confirmed in the studies by Curiel et al. [19]. These researchers also found that Treg cells suppress the response directed against neoplastic cells, which facilitates the growth of a tumor. The mechanism of Treg cells migration was related to the presence of the CCL22 chemokine which is released by the tumor cells and their surrounding macrophages. This chemokine, having bound to the CCR4 receptor, directed migration of CD4 + CD25 + FOXP3+ lymphocytes toward neoplasm [19]. Possibly, the recruitment of Treg lymphocytes toward the tumor is one of the mechanisms of escape of neoplasm from the response of the immune system.

It cannot be excluded that one of the main causes of an increased Treg percentage in the neoplastic microenvironment is their conversion from CD4 + CD25- to CD4 + CD25 + FOXP3+ under the influence of TGF-beta. This hypothesis is in turn confirmed by the studies of Valsazina et al. [20] who found that it is the mechanism of conversion of Treg cells, not their active proliferation in the periphery, which is the cause of their migration towards the tissue of the tumor.

The next part of the experiment was assessment of the influence of Treg lymphocytes on the time of survival of patients. Ovarian cancer patients were divided into two groups depending on the median of the Treg cells percentage. It was found that the survival time of women with a high Treg percentage in peripheral blood (Median >3.7 %) and the tumor tissue (Median >4.6 %) was shorter in opposition to patients with a low percentage of these cells, however, this relationship was not statistically significant. Similar conclusions were drawn by Curiel et al. [19] who found that an increased percentage of Treg lymphocytes was related to a shorter time of patients’ survival. In patients with Hodgkin’s lymphoma Marshall et al. [21] observed an increased percentage of CD4 + CD25+ Treg among lymphocytes infiltrating the lymph nodes as well as in the population of mononuclear blood cells. However, a lower concentration of FOXP3+ cells and a higher concentration of cytotoxic T lymphocytes within the lymph nodes correlated with a shorter time of survival. In the case of Hodgkin’s lymphoma Treg cells inhibit a chronic inflammatory process which is thought to be an important etiopathogenetic factor in lymphomas.

Studies on the murine model concerning the use of anti-CD25(IL2R) antibodies in order to deplete CD4 + CD25+ Treg from peripheral blood are promising. It was observed that their administration resulted in regression of neoplasm, which correlated with a decrease in the percentage of Treg lymphocytes [22]. Moreover, it was shown in this study, conducted by Dannull et al. [22], that the use of recombined IL-2 conjugated with diphtherial toxin DAB(389)IL-2 (denileulkin diftitox called ONTAK) selectively eliminated CD4 + CD25+ Treg cells from peripheral blood, thus strengthening the antineoplastic response. Application of monoclonal antibodies against CD25 (Daclizumab and Basiliximab) in patients with breast carcinoma also strengthened the antineoplastic response [23–25]. Despite the fact that no negative influence was found after using methods of Treg lymphocytes elimination in the studies on mice, attempts at cancer treatment with the use of monoclonal antibodies in people should be made with caution. Improper use of these cells may lead to many undesirable effects and disturbances in the immune response. Therefore, it seems important to broaden knowledge on the subject of mechanisms and effects of action of Treg lymphocytes.

On the basis of the obtained results differences were found in blood serum TGF-β concentrations in patients with malignant and non-malignant tumors. The highest concentration was found in the group of women with cancer of the ovary and it differed significantly from the reference group and the group with benign tumors. No correlation was observed between TGF-beta concentration and the percentage of CD4 + CD25 + FOXP3+ lymphocytes in the microenvironment of the tumor and peripheral blood in individual groups.

TGF-beta may induce suppression of cancer at early stages and at later stages it promotes growth of a tumor. Some authors showed that in benign tumors and early stage of tumors TGF-beta inhibited carcinogenesis and was positively associated with a favorable prognosis. However, in advanced tumors TGF-beta promotes tumor growth and progression [26–28], thanks to which TGF-beta could be a useful prognostic biomarker and predictor of recurrence after initial or failed therapy [29]. Liu et al. used utilized antibodies directed at neutralization of TGF-beta and demonstrated blockade of tumor induced T_reg_ conversion [30]. This could suggest that T_reg_ prevalence in the tumor microenvironment is reduced by blocking TGF-beta and T_reg_ prevalence in the tumor microenvironment is likely influenced by expansion, conversion, and recruitment.

Bartlett et al. [31] estimated that mRNA TGF-beta1 expression was present in 46 among 74 ovarian cancers. It was also demonstrated that TGF-β inhibits proliferation of the ovarian epithelium and induces apoptosis, which may prevent excessive proliferation of cells during ovulation. At an advanced stage of ovarian cancer this cytokine increases growth of neoplastic cells and promotes formation of metastases as a result of transforming epithelial cells into mesenchymal cells [32].

Converting CD4 + CD25- cells into CD4 + CD25+ cells TGF-beta induces in them the FOXP3 gene expression. It was shown that most CpG islets of the FOXP3 gene promoter in CD4 + CD25- lymphocytes have methylic groups, whereas in CD4 + CD25+ cells nearly all are demethylated [33–35]. CD4 + CD25+ cells exhibit constant FOXP3 expression, CD4 + CD25- do not show constitutive FOXP3 expression. The mechanism through which TGF-beta affects an increase in the FOXP3 gene expression consists in acetylation of histones in the region of the transcription intensifier and demethylation of CpG islets in the first intron of the FOXP3 gene [34, 35]. Moreover, the evidence to support the theory of conversion of CD4 + CD25- into CD4 + CD25+ and expression of FOXP3 mediated by TGF-beta is that Smad3 – a transcription factor activated by TGF-beta binds to the transcription intensifier, playing a key role in the increase in the FOXP3 gene expression [36].

Transmission of signals by TGF is important for transformation of neoplastic cells. Therefore, modulation of signals by TGF may play a role in the therapy of neoplasms. TGF AP 12009 inhibitors were used in phase I/II of clinical studies in the treatment of advanced pancreatic cancer and other neoplasms [37]. LY2109761, an inhibitor of TGF receptors inhibited metastases of cancer of the pancreas [38]. It was demonstrated in the preclinical model that anti-TGF antibody decreased the number of Treg cells in neoplastic tumors and the lymph nodes. This effect was strengthened by a blockade of B7-H1(CD28-CD80) connection [39]. Thus, it is obvious that blocking TGF transmission can affect the percentage and role of Treg lymphocytes.

In our study we find a higher percentage of T_reg_ in malignant tissue compared to benign disorders. It is well known that T_reg_ lymphocytes decrease efficacy of cancer immunotherapy through their high immunosuppressive potential. An increased level of regulatory T cells could be associated with the function of the ovarian tumor macrophages which promote T_reg_ trafficking to the tumor. This immunosuppressive action could be overcome by blocking CCL22 and/or B7-H4 molecules, which helps to potentiate T cell antitumor responses [19, 40]. In 2011 a humanized anti-CSF-1R mAb, RG7155 (Roche) entered clinical trials. The results from the ongoing Phase Ia/Ib clinical trial (NCT01494688) indicate that RG7155 treatment is well tolerated and effectively depletes macrophages associated with a tumor, which results in a decreased number of FOXP3 positive T_reg_ lymphocytes [41]. Targeting macrophages is a promising therapeutic approach to ovarian cancer and early encouraging work indicates that CSF-1R blockade, anti-B7-H4 scFvs and anti-CCL22 mAbs may generate potent antitumor responses, which is associated with elimination of regulatory T cells. Indoleamine 2,3-dioxygenase (IDO) is the leading metabolic immune regulator which increases expression of FOXP3 in T cells. In ovarian cancers, immunohistochemical examination of surgically resected tumors has demonstrated that IDO is prevalent in ~56 % of ovarian tumors and correlates with a reduced antitumor immune response [42]. Currently, the IDO inhibitors: Indoximod® (NewLink Genetics), NLG919 (NewLink Genetics) and INCB024360 (Incyte Corp.) entered clinical trials [41]. All of them showed the ability to potentiate immune response and it could be associated with diminishing of T_reg_. In our work we find a statistically significant increase in T_reg_ cells in malignant tissue, which is in accordance with findings confirming the key role of these cells in cancer induced immunosuppression, that could be overcome with immunotherapies targeting different stages in T_reg_ development and function. However, a higher percentage of T_reg_ was not significantly correlated with shorter survival of patients, probably due to a small number of patients included in the study.

T_reg_ cells can have a drawback, which means that their large numbers will lead to cancer. Patients with tumors have a local excess of T_reg_ lymphocytes. Their systemic levels likely influence levels in cancer tissues. Our conclusions are that patients with ovarian cancer have elevated levels of serum TGF-beta and T_reg_ numbers are elevated in the tumor microenvironment of patients with ovarian cancer. This suggests that T_reg_ within the tumor microenvironment may play a significant role in suppressing antitumor immunity.

## Conclusions

The recruitment of T_reg_ lymphocytes toward the tumor is one of the mechanisms of escape of neoplasm from the response of the immune system. The percentage of T_reg_ lymphocytes in peripheral blood and the tumor tissue does not influence the time of survival of patients with ovarian cancer.

## Abbreviations

DMSO: 10 % dimethyl sulphoxide
FCS: Fetal calf serum
FOXP3: (*forkhead box P3)* transcription factor
IDO: Indoleamine 2,3-dioxygenase
PBMC: Peripheral blood mononuclear cells
PBS: Physiological saline solution
TGF-β: Transforming growth factor-beta
T_reg_: Regulatory T cells

## References

[1] Sakaguchi S, Sakaguchi N, Asano M, Itoh M, Toda M (1995). Immunologic self-tolerance maintained by activated T cells expressing IL-2 receptor alpha-chains (CD25). Breakdown of a single mechanism of self-tolerance causes various autoimmune diseases. J Immunol.

[2] Fontenot JD, Rudensky AY (2005). A well adapted regulatory contrivance: regulatory T cell development and the forkhead family transcription factor Foxp3. Nat Immunol.

[3] Wan YY, Flavell RA (2008). TGF-β and regulatory t cell in immunity and autoimmunity. J Clin Immunol.

[4] Baecher-Allan C, Brown JA, Freeman GJ, Hafler DA (2001). CD4 + CD25^high^ regulatory cells in human peripheral blood. J Immunol.

[5] Thompson C, Powrie F (2004). Regulatory T cells. Curr Opin Pharmacol.

[6] Kotake S, Udagawa N (1999). Rheumatoid arthritis is a potent stimulator of osteoclastogenesis. J Clin Invest.

[7] Nakamura K, Kitani A, Strober W (2001). Cell contact-dependent immunosuppression by CD4(+)CD25(+) regulatory T cells is mediated by cell surface-bound transforming growth factor β. J Exp Med.

[8] Piccirillo CA, Letterio JJ, Thornton AM, McHugh RS, Mamura M, Mizuhara H, Shevach EM (2002). CD4(+)CD25(+) regulatory T cells can mediate suppressor function in the absence of transforming growth factor β1 production and responsiveness. J Exp Med.

[9] Andersson J, Tran DQ, Pesu M, Davidson TS (2008). CD4+ FoxP3+ regulatory T cells confer infectious tolerance in a TGF-beta-dependent manner. J Exp Med.

[10] Lin Y, Kikuchi S, Tamakoshi A (2006). Serum transforming growth factor-beta1 levels and pancreatic cancer risk: a nested case–control study (Japan). Cancer Causes Control.

[11] https://www.ovariancancer.org/about/statistics/.

[12] Scully RE, Young RH, Clement PB (1998). Tumors of ovary, mal developed gonads, fallopian tube, and broad ligament. In: Atlas of tumor pathology.

[13] Chen VW, Ruiz B, Killeen JR, Cote’ TR, Wu XC, Correa CN (2003). Pathology and classification of ovarian tumors. Cancer.

[14] Pettersson F (1991). Annual report of the results of treatment in gynecological cancer.

[15] Averette HE, Janicek MF, Menck HR (1995). The national cancer data base report on ovarian cancer. American college of surgeons commission on cancer and the american cancer society. Cancer.

[16] Woo EY, Chu CS, Goletz TJ, Schlienger K, Yeh H, Coukos G, Rubin SC, Kaiser LR, June CH (2001). Regulatory CD4 + CD25+ T cells in tumors from patients with early-stage non-small cell lung cancer and late-stage ovarian cancer. Cancer Res.

[17] Fialová A, Partlová S, Sojka L, Hromádková H, Brtnický T, Fučíková J, Kocián P, Rob L, Bartůňková J, Spisek R (2013). Dynamics of T-cell infiltration during the course of ovarian cancer: the gradual shift from a Th17 effector cell response to a predominant infiltration by regulatory T-cells. Int J Cancer.

[18] Liyanage UK, Moore TT, Joo HG, Tanaka Y, Herrmann F, Doherty G, Drebin JA, Strassberg SM, Eberlein TJ (2002). Prevalence of regulatory T cells is increased in peripheral blood and tumor microenvironment of patients with pancreas or breast adenocarcinoma. J Immunol.

[19] Curiel TJ, Coukos G, Zou L, Alvarez X, Cheng P, Mottram P, Evdemon-Hogan M, Conejo-Garcia JR, Zhang L, Burow M, Zhu Y, Wei S, Kryczek I, Daniel B, Gordon A, Myers L, Lackner A, Disis ML, Knutson KL, Chen L, Zou W (2004). Specific recruitment of regulatory T cells in ovarian carcinoma fosters immune privilege and predicts reduced survival. Nat Med.

[20] Valzasina B, Piconese S, Guiducci C, Colombo MP (2006). Tumor-induced expansion of regulatory t cells by conversion of CD4 + CD25- lymphocytes is thymus and proliferation independent. Cancer Res.

[21] Marshall NA, Christie LE, Munro LR, Culligan DJ, Johnston PW, Barker RN, Vickers MA (2004). Immunosuppressive regulatory T cells are abundant in the reactive lymphocytes of Hodgkin lymphoma. Blood.

[22] Dannull J, Su Z, Rizzieri D, Benjamin K, Yang, Coleman D, Yancey D, Zhang A, Dahm P, Chao N, Gilboa E, Vieweg J (2005). Enhancement of vaccine-mediated antitumor immunity in cancer patients after depletion of regulatory T cells. J Clin Invest.

[23] Nashan B, Moore R, Amlot P, Schmid A, Abeywickrama K, Soulillou J (1997). Randomised trial of basiliximab versus placebo for control of acute cellular rejection in renal allograft recipients. Lancet.

[24] Vincenti F, Kirkman R, Light S, Bumgardner G, Pescovitz M, Halloran P, Neylan J, Wilkinson A, Ekberg H, Gaston R, Backman L, Burdick J (1998). Interleukin-2-receptor blockade with daclizumab to prevent acute rejection in renal transplantation. N Engl J Med.

[25] Rech A, Vonderheide R (2009). Clinical use of anti-CD25 antibody daclizumab to enhance immune responses to tumor antigen vaccination by targeting regulatory T cells. Ann N Y Acad Sci.

[26] Padua D, Massague J (2009). Roles of TGF beta in metastasis. Cell Res.

[27] Inman GJ (2011). Switching TGF, beta from a tumor suppressor to a tumor promoter. Curr Opin Genet Dev.

[28] Wendt MK, Tian M, Schiemann WP (2012). Deconstructing the mechanisms and consequences of TGF-beta-induced EMT during cancer progression. Cell Tissue Res.

[29] Langenskiold M, Holmdahl L, Falk P (2008). Increased TGF-beta 1 protein expression in patients with advanced colorectal cancer. J Surg Oncol.

[30] Liu VC, Wong LY, Jang T (2007). Tumor evasion of the immune system by converting CD4 + CD25− T cells into CD4 + CD25+ T regulatory cells: role of tumor-derived TGF-beta. J Immunol.

[31] Bartlett JM, Langdon SP, Scott WN, Love SB, Miller EP, Katsaros D, Smyth JF, Miller WR (1997). Transforming growth factor-beta isoform expression in human ovarian tumours. Eur J Cancer.

[32] Choi KC, Kang SK, Tai CJ, Auersperg N, Leung PC (2001). The regulation of apoptosis by activin and transforming growth factor-beta in early neoplastic and tumorigenic ovarian surface epithelium. J Clin Endocrinol Metab.

[33] Floess S, Freyer J, Siewert C, Baron U, Olek S, Polansky J, Schlawe K, Chang HD, Bopp T, Schmitt E, Klein-Hessling S, Serfling E, Hamann A, Huehn J (2007). Epigenetic control of the foxp3 locus in regulatory T cells. PLoS Biol.

[34] Ichihara F, Kono K, Takahashi A, Kawaida H, Sugai H, Fujii H (2003). Increased populations of regulatory T cells in peripheral blood and tumor-infiltrating lymphocytes in patients with gastric and esophageal cancers. Clin Cancer Res.

[35] Polansky JK, Kretschmer K, Freyer J, Floess S, Garbe A, Baron U, Olek S, Hamann A, von Boehmer H, Huehn J (2008). DNA methylation controls foxp3 gene expression. Eur J Immunol.

[36] Xu L, Kitani A, Strober W (2010). Molecular mechanisms regulating tgf-beta-induced foxp3 expression. Mucosal Immunol.

[37] Schlingensiepen H, Fischer-Blass B, Schmaus S, Ludwig S (2009). Antisense therapeutics for tumor treatment: the TGF-beta2 inhibitor AP 12009 in clinical development against malignant tumors. Recent Res Cancer.

[38] Melisi D, Ishiyama S, Sclabas G, Fleming JB, Qia Q, Tortora G, Abbruzzese JL, Chiao PJ (2008). LY2109761, a novel transforming growth factor β receptor type I and type II dual inhibitor, as a therapeutic approach to suppressing pancreatic cancer metastasis. Mol Cancer Ther.

[39] Søndergaard H, Skak K (2009). IL-21: roles in immunopathology and cancer therapy. Tissue Antigens.

[40] Dangaj D, Lanitis E, Zhao A, Joshi S, Cheng Y, Sandaltzopoulos R, Ra HJ, Danet-Desnoyers G, Powell DJ, Scholler N (2013). Novel recombinant human b7-h4 antibodies overcome tumoral immune escape to potentiate T-cell antitumor responses. Cancer Res.

[41] Chester C, Dorigo O, Berek JS, Kohrt H (2015). Immunotherapeutic approaches to ovarian cancer treatment. J Immunother Cancer.

[42] Inaba T, Ino K, Kajiyama H, Yamamoto E, Shibata K, Nawa A, Nagasaka T, Akimoto H, Takikawa O, Kikkawa F (2009). Role of the immunosuppressive enzyme indoleamine 2,3-dioxygenase in the progression of ovarian carcinoma. Gynecol Oncol.

